# Air-Filled Microbubbles Based on Albumin Functionalized with Gold Nanocages and Zinc Phthalocyanine for Multimodal Imaging

**DOI:** 10.3390/mi12101161

**Published:** 2021-09-27

**Authors:** Elizaveta A. Maksimova, Roman A. Barmin, Polina G. Rudakovskaya, Olga A. Sindeeva, Ekaterina S. Prikhozhdenko, Alexey M. Yashchenok, Boris N. Khlebtsov, Alexander A. Solovev, Gaoshan Huang, Yongfeng Mei, Krishna Kanti Dey, Dmitry A. Gorin

**Affiliations:** 1 Center for Photonics and Quantum Materials, Skolkovo Institute of Science and Technology, 3 Nobelya Str., 121205 Moscow, Russia; Elizaveta.Maksimova@skoltech.ru (E.A.M.); Roman.Barmin@skoltech.ru (R.A.B.); P.Rudakovskaya@skoltech.ru (P.G.R.); A.Yashchenok@skoltech.ru (A.M.Y.); 2 Center for Neurobiology and Brain Restoration, Skolkovo Institute of Science and Technology, 3 Nobelya Str., 121205 Moscow, Russia; O.Sindeeva@skoltech.ru; 3 Science Medical Center, Saratov State University, 83 Astrakhanskaya Str., 410012 Saratov, Russia; prikhozhdenkoes@gmail.com; 4 Institute of Biochemistry and Physiology of Plants and Microorganisms, 410049 Saratov, Russia; khlebtsov_b@ibppm.ru; 5 Department of Materials Science, Fudan University, Shanghai 200433, China; solovevlab@gmail.com (A.A.S.); gshuang@fudan.edu.cn (G.H.); yfm@fudan.edu.cn (Y.M.); 6 Discipline of Physics, Indian Institute of Technology Gandhinagar Gandhinagar, Gujarat 382355, India; k.dey@iitgn.ac.in

**Keywords:** microbubbles, bovine serum albumin, gold nanocages, zinc phthalocyanine, ultrasound imaging, fluorescence tomography, optoacoustic imaging, multimodal imaging

## Abstract

Microbubbles are intravascular contrast agents clinically used in diagnostic sonography, echocardiography, and radiology imaging applications. However, up to date, the idea of creating microbubbles with multiple functionalities (e.g., multimodal imaging, photodynamic therapy) remained a challenge. One possible solution is the modification of bubble shells by introducing specific compounds responsible for such functions. In the present work, air-core microbubbles with the shell consisting of bovine serum albumin, albumin-coated gold nanocages, and zinc phthalocyanine were prepared using the sonication method. Various physicochemical parameters such as stability over time, size, and concentration were investigated to prove the potential use of these microbubbles as contrast agents. This work shows that hybrid microbubbles have all the necessary properties for multimodal imaging (ultrasound, raster-scanning microscopy, and fluorescence tomography), which demonstrate superior characteristics for potential theranostic and related biomedical applications.

## 1. Introduction

Nowadays, biomedical ultrasound (US) imaging is one of the most frequently used clinically available methods; it has been widely used for diagnostics for more than 40 years [1,2]. The first US contrast agent was discovered back in the last century when Gramiak and Shah observed enhanced echogenicity of the aortic root after rapid injection of saline [3]. Later, this observation was attributed to the presence of microbubbles occurring in the bloodstream after injection [4]. Subsequently, it was found that microbubbles with a diameter larger than 7 μm could not be transported through lung capillaries. These bubbles, with sizes comparable to red blood cells, flow without being phagocytized and adversely affect the blood pool [5,6]. 

Over recent decades, plenty of microbubbles-based agents have been widely investigated and involved in clinical practice as US contrast agents due to their excellent acoustic properties [2,7,8]. The potential use of microbubbles is mainly determined by their structure and stability, which can be altered by their core and shell compositions. The first-generation of ultrasound contrast agents was presented mainly as bubbles filled with either air or gases with similar density (oxygen, nitrogen, etc.) [9,10,11]. Due to the relatively high solubility of air components in physiological fluids, the use of such bubbles was limited by their short lifetime in the bloodstream [12]. The second generation of microbubbles is presented with sulfur hexafluoride or perfluorocarbons as gases for the core since the high molecular weight and low solubility of these gases in water lead to a noticeable increase in echogenicity and circulation time in the bloodstream [5]. However, an equally important task of microbubble engineering is the choice of material for bubbles shell preparation. The shell can consist of a wide range of bioactive compounds: proteins, lipids, or even synthetic surfactants [13,14,15]. The chemical composition of the microbubble shell and the bonds between the shell components significantly affect the stability of contrast agents, which is a crucial parameter for bio-applications. Polymers’ use leads to the formation of a rigid shell, the apparent disadvantage of which is the low amplitude of oscillation under the ultrasound impact and, as a consequence, the decrease in acoustic responses [16]. Moreover, microbubbles based on surfactants or phospholipids possess a softer shell. The encapsulated gas can diffuse through the shell into the surrounding fluid, which significantly reduces the stability of microbubbles in time [17].

A reasonable number of articles are dedicated to protein shell microbubbles [11,13,18,19,20,21,22,23]. The advantages of using proteins are their high biocompatibility and optimal packing density in the shell [24]. In the present work, bovine serum albumin (BSA) was chosen as the main shell component since BSA structure and properties are very similar to those of human serum albumin that is present in the blood plasma in sufficient concentration. Therefore, using BSA microbubbles causes fewer immune responses; namely, allergic reactions may rarely be observed [13]. Previously, two types of commercially available echocardiographic US contrast agents based on human serum albumin were fabricated. Albunex (Molecular Biosystems Inc., San Diego, CA, USA) represents sonicated 5% human serum albumin and shows no major or minor adverse effects [25]. Optison (GE Healthcare AS, Oslo, Norway) also showed negligible adverse effects evaluated by 6.5% [26]. Although albumin performs as a promising basis for contrast agents’ production, multimodality and multifunctionality properties have not been studied in detail.

A commonly used method for microbubbles production is the ultrasonic treatment (known as the sonication method) of a system containing a shell material and a gas for encapsulation [13,22,27]. The mechanism of forming the protein shell of microbubbles under sonication includes the destruction of intramolecular disulfide bonds between cysteine-containing amino acids when heated up to temperatures close to denaturation [28]. The resulting free thiol groups form intermolecular disulfide bridges stabilizing the microbubble shell during the ultrasound impact [29]. Additionally, it was suggested that the reactive oxygen species formed during acoustic/ultrasonic cavitation act as primary oxidants and are responsible for the crosslinking of the thiol groups [30].

In addition to implementing microbubbles for US imaging, there is an urgent need for agent modification with functional additives aiming at multimodal imaging and/or theranostic purposes. Additional molecules or substances should be introduced in the structure of the microbubble shell to achieve such modalities. Previously, the possibility of a contrast-enhanced combination of photoacoustic (PA) and US imaging was acquired by the inclusion of gold nanoparticles [31]. Their outstanding optical characteristics, in general, and morphology-dependent plasmonic properties, in particular, allow selecting the conditions for achieving the highest temporal and spatial resolution of the obtained image [32,33]. Mohamedi et al. described theoretically and proved experimentally that the microbubbles prepared from a liquid suspension of gold nanoparticles were found to have significantly enhanced stability over time and size compared with bubbles coated only with surfactant [34]. Furthermore, by functionalizing the surface of gold nanoparticles with specific ligands, microbubbles-based contrast agents can target certain organs or tissues, offering opportunities to monitor the course of various diseases and disorders [35].

Recently, our group obtained multimodal microbubbles for PA/fluorescent (FL)/US imaging. PA properties are achieved by introducing gold nanorods into the surfactants-stabilized microbubbles and introducing gold nanospheres into the protein shell structure of bubbles [14,18]. In this work, we use gold nanocages (AuNCs) due to their high extinction coefficients in a “biological window” (650–1350 nm), so they can also be exploited in PA imaging [36]. AuNCs were already reported in PA imaging in order to detect B16 melanomas at 778 nm and U87 brain tumors [37]. Additionally, gold nanocages were found to be considerably active for plasmonic photothermal therapy of prostate tumor [38] and breast cancer cells in vitro [39]. However, to the best of our knowledge, they have never been incorporated in the microbubbles shell for the same purposes.

Furthermore, theranostics is nowadays a rapidly developing scientific field, where imaging technologies often involve smart therapeutic strategies. Concepts such as molecular imaging actively involve the usage of bubble-micromotors with shell modification for cancer treatment [40]. Photodynamic therapy (PDT) is a non-invasive cancer treatment method well-established in clinical practice over the decades. The procedure is based on the system that comprises the photosensitizing chemical compound and molecular oxygen located in tissue and the laser beam’s use with an appropriate wavelength [41,42]. The reactive oxygen species are produced under photon-induced excitation initiated by laser radiation, resulting in cell death. One promising substance that can act as photosensitizers is phthalocyanines, aromatic heterocycles consisting of four isoindole rings bridged by nitrogen atoms. One of the most popular PDT dyes of this group is aluminum phthalocyanine, known under the trade name Photosens, which is approved for medical use in the Russian Federation and is widely used in clinical practice [43]. Along with zinc phthalocyanine, known as Holosens, which is under preclinical trials in Russia [44], those dyes have become an object of growing interest in PDT application for oncology and infectious diseases in dentistry, otorhinolaryngology, gynecology, and urology [43,45,46,47]. Previously, the possibility of the introduction of fluorescent dyes into the albumin structure was investigated for further formation of microbubbles with modified shells [18]. The combination of photodynamic dyes with microbubbles-assisted imaging opens new horizons in the field of multifunctional theranostics. In this work, zinc phthalocyanine (ZnPc) is used as a photosensitizer due to the high extinction coefficients and high fluorescence quantum yield [48].

In the present work, trimodal microbubbles-based US/PA contrast agents with the BSA shell are presented with the potential to be used in the PDT procedure. As functional additives, gold nanocages (AuNCs) were used with BSA-shell coating providing the possibility of PA imaging, as well as the photodynamic dye, zinc phthalocyanine (ZnPc), in conjugation with BSA for optimal incorporation in the microbubbles shell structure, providing FL imaging purposes and the potential for PDT cancer treatment. To the best of our knowledge, hybrid systems of such composition have never been reported elsewhere. Thus, our paper is focused on microbubbles synthesis and characterization for further possible biomedical applications. We reveal storage stability, mean sizes, and concentration measurements of obtained agents over time and physicochemical characterization. The obtained modalities of the microbubble shell were probed by different methods of analysis, namely UV-VIS spectroscopy, fluorescence tomography, optoacoustic, and ultrasound imaging.

## 2. Materials and Methods

### 2.1. Materials

Bovine serum albumin (BSA), Chloroauric acid (HAuCl_4_·3H_2_O), Hexadecyltrimethylammonium bromide (CTAB) (C_19_H_42_BrN), Hexadecyltrimethylammonium chloride (CTAC) (C_19_H_42_ClN), ascorbic acid (AA), Sodium borohydride (NaBH_4_), Silver nitrate (AgNO_3_), sodium citrate (Na_3_C_6_H_5_O_7_·3H_2_O), phosphate buffer saline (PBS), *N*-(3-Dimethylaminopropyl)-*N*′-ethyl carbodiimide hydrochloride (EDC), *N*-Hydroxysuccinimide (NHS), Sodium chloride (NaCl), and agarose were all purchased from Sigma-Aldrich (Germany). Holosens^®^, octachloride octakis [N (2-hydroxyethyl) -*N*,*N*, -(dimethylammoniomethyl)] zinc (II) phthalocyanine (ZnPc) was chosen as a photodynamic dye and obtained from Organic Intermediates and Dyes Institute (Russia). Deionized (DI) water with specific resistivity, higher than 18.2 MΩm from a Milli-Q Integral 3 water purification system (Millipore, MA, USA), was used to make all solutions.

### 2.2. Methods

#### 2.2.1. AuNCs Synthesis

The AuNCs were prepared using a seed-mediated multistep procedure. First, 10 nm monodisperse Au seeds were prepared as described elsewhere [49]. To this end, Au clusters were prepared by mixing 0.25 mL of 10 mM HAuCl_4_ into 10 mL of 0.1 M aqueous CTAB and 0.6 mL of a 10 mM NaBH_4_ solution. Then, 10 nm Au seeds were prepared by mixing 20 mL of 0.1 M CTAC, 15 mL of 0.1 M AA, 0.5 mL of nanoclusters, and 20 mL of 0.5 mM HAuCl4. Second, 100 µL of seeds were added to 8 mL of 20 mM CTAC followed by 800 µL of 100 mM ascorbic acid and 200 µL of 100 mM AgNO3. The reaction was allowed to proceed for 3 h at 70 °C without stirring. The resulting solution has a bright yellow color, which indicates the formation of 40 nm Ag cubes with a high yield. The resulting nanoparticles were centrifuged (8000 *g*, 20 min) and resuspended in 10 mL of 50 mM CTAB. This procedure was repeated thrice. Third, Ag nanocubes were converted to Au nanocages by using a galvanic replacement reaction. To this end, 500 µL of 10 mM HAuCl4 were added to 10 mL of Ag nanocubes dispersed in 50 mM CTAB. The reaction was allowed to proceed for 3 h at room temperature (RT) without stirring. During this period, the suspension color gradually changed from yellow to orange, red, purple, blue, and finally blue-green. The resulting Au nanocages were centrifuged (8000 *g*, 20 min) and resuspended in water. Note, our original procedure of galvanic replacement between Ag atoms and Au3+-CTAB complex has several advantages compared to the well-known protocols [50]. The proposed reaction proceeds at room temperature and does not require the gradual addition of reagents at a controlled rate. Additionally, the obtained particles do not need additional purification from silver chloride and polyvinylpyrrolidone.

The coating of obtained AuNCs was performed according to the technique developed by the authors [51] and modified in our group. Briefly, for replacing CTAB with BSA, the AuNCs solution is added dropwise into the solution containing BSA 10 mg/mL, sodium citrate 0.1% in volume ratio 1:1 (pH = 12). The resulting solution is treated in an ultrasonic bath for 30 min. After that, the nanoparticles are centrifuged at 14,000 rpm for 30 min, washed with the same replacing solution three times, and resuspended in the BSA water solution (1 mg/mL, pH = 7). For further use, solutions were concentrated 4 times.

#### 2.2.2. ZnPc-BSA Conjugate Preparation

The synthesis of the crosslinked conjugate of BSA and ZnPc was carried out by the carbodiimide method. To do this, 30 mg of BSA is dissolved in 7 mL of phosphate buffer (PBS) with pH = 8775 μL of 1 mg/mL EDC solution in PBS is added under stirring. The resulting solution is kept under stirring for 15 min, then 1.05 mL of 1 mg/mL NHS solution in PBS is added and stirred for another 15 min. After adding 1 mL of 1 mg/mL Holosens, the solution is left under stirring for at least 3 h in cold conditions (4 °C). The solution is purified by 12 h long dialysis against DI water and stored at 4 °C.

#### 2.2.3. Mass Spectrometry Measurements

The efficiency of ZnPc-BSA conjugation was studied using a time-of-flight mass spectrometer with matrix laser desorption/ionization (MALDI-TOF/TOF) rapifleX MALDITOF/TOF MS System (Bruker Daltonik GmbH, Bremen, Germany). The operating mode was the following: linear mode, positive ionization, analysis range *m*/*z* 5000–70,000, accelerating voltage 20 kV, SmartBeam III laser, laser frequency 10 kHz, frequency 200 Hz. Before analysis, the device was calibrated using a mixture of proteins “Protein Calibration Standard I” (Bruker Daltonik GmbH, Germany). The mixture included the following proteins: insulin ([M+H] = *m*/*z* 5734.5), ubiquitin I ([M + H] = *m*/*z* 8565.76), cytochrome C ([M+H] = *m*/*z* 12,361.2), myoglobin ([M+H] = *m*/*z* 16,952.5). 2.5-dihydroxybenzoic acid (Bruker Daltonik GmbH, Germany) with purity > 99.0% was used as the matrix. A 20 mg/mL matrix solution was prepared in a mixture of 30% acetonitrile: 70% water: 0.1% trifluoroacetic acid. Aqueous solutions of the samples were mixed with the matrix in a ratio of 1:1, and 1 μL of the mixture was applied to the plate.

#### 2.2.4. Dynamic Light Scattering and Z-Potential Measurements

DLS and zeta-potential measurements were performed on the ZetaSizer Nano ZS analyzer (Malvern Panalytical, Malvern, UK). For zeta-potential measurements, all samples were diluted 20 times in DI water and placed in a U-shape cuvette; for DLS measurements, all samples were diluted 40 times in DI water and placed in the plastic cuvette. Results were processed by Zetasizer Software 8.00. Each measurement was carried out at 25 °C and repeated three times.

#### 2.2.5. Nanoparticles Tracking Analysis

For NTA characterization, solutions of AuNCs and AuNCs-BSA were diluted 200 times with DI water. The measurements were performed on the NanoSight model NS300 (Malvern, Salisbury, UK). The diluted sample was injected into the test cell and exposed to 45 mW laser of wavelength 488 nm. The scattered light was video recorded using the built-in high sensitivity sCMOS camera for 60 s at 25 frames per second with 0.1 ms shutter speed. For each sample, the recording was repeated 5 times, and each video consisted of 1498 frames that captured at least 2000 valid particle tracks. The videos were analyzed by NTA software (Version 3.2) to produce the size distribution and concentration of nanoparticles. The following parameters were used during the analysis: the minimum track length and blur size were set to auto, and the detection threshold was set to 30. The cell’s temperature was measured automatically by the NTA instrument and maintained at level 296.15−297.15 K during room temperature measurements. The viscosity of DI water at room temperature is nearly constant and equal to ~0.91 cP.

#### 2.2.6. Microbubbles Preparation

Microbubbles were obtained by the modified sonication method. Briefly, for each sample, 150 mg of BSA were dissolved in 1 mL of 2.7% NaCl aqueous solution to produce each microbubbles sample from the components dissolved in saline solution. Then, 1 mL of ZnPc-BSA solution was added in each sample of microbubbles labeled with ZnPc (BSA-ZnPc MBs, BSA-Au-ZnPc MBs); then, for the sample containing AuNCs (BSA-Au-ZnPc MBs), 1 mL of AuNC-BSA solution was added, while for sample labeled with ZnPc only (BSA-ZnPc MBs), 1mL of DI water was added. Likewise, for the sample containing gold nanoparticles only (BSA-Au MBs), 1 mL of AuNC-BSA solution and 1 mL of DI water were added. For the preparation of microbubbles with the BSA shell without any additives, 2 mL of DI water was added to 1 mL of BSA solution obtained as described before. All samples were placed in a glass vial and heated to a temperature of 50 °C in order to lower the solution’s surface tension and promote partial denaturation of BSA. After several minutes of incubation, each sample was sonicated for 5 min at the maximum power of 100 W on the Bandelin Sonopuls HD4100 sonicator with the TS103 sonotrode probe (Bandelin Electronic GmbH & Co KG, Berlin, Germany). The tip of the sonotrode was placed at the liquid–air interface. After sonication, each sample was stored at 4 °C for 30 min for further stabilization. All obtained samples with microbubbles were dialyzed at 4 °C for 12 h against a saline solution in order to purify resulting microbubbles from unreacted initial compounds.

#### 2.2.7. Optical Microscopy for Concentration and Size Measurements

Optical microscopy (OM) was carried out on Olympus CX33 (Olympus Corporation, Tokio, Japan). The size distribution of microbubbles was evaluated using images of 200 bubbles.

Microbubbles’ concentration was determined using Gorjaev’s chamber. Briefly, 10 μL of microbubbles solution before and after dialysis (5 or 10 times diluted) were injected between the glass slides, then stored at room temperature for 5 min in order for the bubbles to float to the upper glass slide. Then, photographs were carried out with the optical microscope within the grid of the chamber. For each sample, more than 200 microbubbles were counted to determine the concentration of samples. For each microbubble probe, concentrations were evaluated 30 min after the sonication (further labeled as 1 h), after storage at 4 °C for 12 h before and after dialysis, and after storage at 4 °C for 36 h before and after dialysis. Each measurement was repeated 5 times.

#### 2.2.8. Transmission Electron Microscopy

Transmission electron microscopy (TEM) images were obtained on a Zeiss M912 Omega transmission electron microscope (Carl Zeiss Microscopy GmbH, Oberkochen, Germany) at an operating voltage of 300 kV.

#### 2.2.9. Extinction Spectra Measurements

Extinction spectra measurements were performed by a multifunctional microplate reader Tecan Infinite M Nano+ (Tecan Trading AG, Männedorf, Switzerland) at room temperature (25 °C). For that, 150 μL of sample solution was placed in a separate well of a plastic 96-well plate. All samples were diluted in saline with concentrations of 100, 50, 25, 12, and 6.5 million bubbles per mL. For each sample, three parallel measurements were provided to improve the reliability of the results.

#### 2.2.10. Fluorescence Tomography Measurements

For fluorescence tomography measurements, each sample was diluted in saline and added to a 96 well plate in the same manner as for extinction spectra measurements. The plate with samples was then imaged by the IVIS CT Spectrum In Vivo system (Xenogen Corp., Alameda, CA, USA) at room temperature (25 °C). Sequence images were acquired with the Excitation/Emission pair of 675/720 nm. Exposure time is auto, FOV = C. Photons were quantified with the LivingImage software (Xenogen Corp., Alameda, CA, USA).

#### 2.2.11. Raster-Scanning Optoacoustic Mesoscopy

To collect optoacoustic signals from samples, a raster-scanning optoacoustic mesoscopy system (RSOM) Explorer P50 (iThera-Medical GmbH, Munich, Germany) was used. The optoacoustic signals were collected by a custom-made spherically focused LiNbO3 detector with the following parameters: center frequency—50 MHz; bandwidth—11–99 MHz; focal diameter—3 mm; focal distance—3 mm. The samples were irradiated by a frequency-doubled flashlamp-pumped Nd:YAG laser (532 nm, pulse duration—2.5 ns; laser pulse energy—200 μJ; repetition rate—1–2 kHz). Light from the laser was delivered through a glass fiber 2-arm bundle (spot size—3.5–5 mm). The scanning head was mounted to 2 motorized stages (field view up to 12 × 12 × 4 mm). The samples were tested in the agarose phantom, for the preparation of which 100 mg of agarose was dissolved in 10 mL of DI water at room temperature. After that, the solution was boiled at 100 °C under intensive agitation and then degassed to prevent the small air bubbles formation in the phantom. For a phantom formation, a droplet of agarose (30 μL) was placed on the bottom of the reservoir, and then after 15 s, a droplet of a sample (7 μL) was injected into the upper third of the formed agarose phantom. Additionally, phantoms with a liquid reservoir of sample inside were stored at 4 °C for 15 min to solidify the phantom, and then the phantom with the sample was covered with a layer of DI water (1.5 cm) to carry out the measurements. The scan head was coupled to the sample via a water-filled reservoir, and the samples were scanned over the 8 × 8 mm field of view with the predefined depth of 4 mm.

#### 2.2.12. Ultrasound Characterization

The DUB^®^ Skinscanner (Taberna Pro Medicum GmbH, Lueneburg, Germany) was used to evaluate ultrasound contrast of obtained microbubbles with a 33 MHz applicator (depth of scanning 8 mm, axial resolution 42 μm). The received signals from the applicator were processed using DUB SkinScanner software (Taberna Pro Medicum GmbH, Lueneburg, Germany).

## 3. Results and Discussion

### 3.1. Functional Additives Characterization for Multimodality Implementation

**Figure 1:**
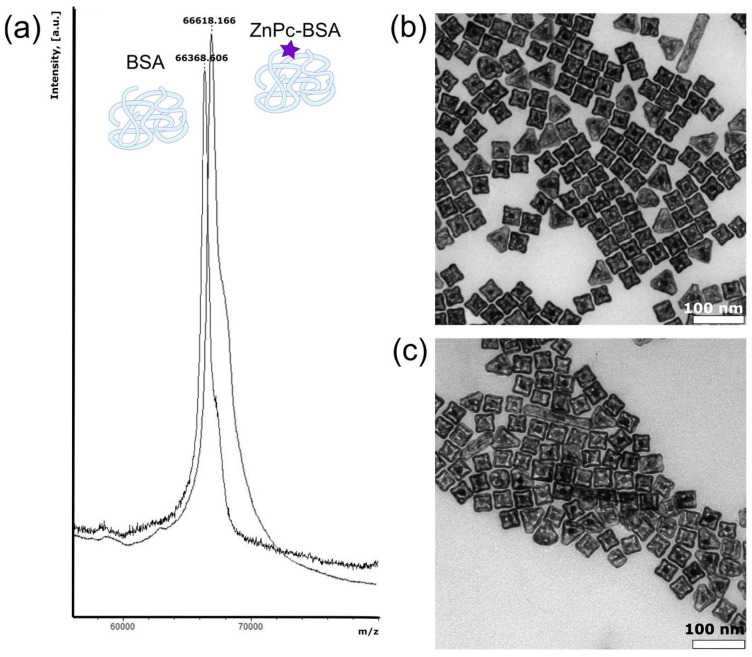
(**a**) Mass spectrometry measurement for BSA aqueous solution and an aqueous solution of ZnPc-BSA conjugate; (**b**) TEM image of gold nanocages (AuNCs); (**c**) TEM image of BSA-coated gold nanocages (AuNC-BSA).

To implement FL imaging modality and the usage of microbubbles in photodynamic therapy (PDT), photodynamic dye zinc phthalocyanine (ZnPc) was covalently bound to BSA via carbodiimide synthesis for conjugate formation. ZnPc was chosen as a model photosensitizer due to the high extinction coefficients and high fluorescence quantum yield [48]. The covalent nature of forming bonds was confirmed by mass spectrometry measurements; the results are shown in Figure 1a. As it can be seen, the BSA peak shifts to ZnPc molecular weight in the case of ZnPc-BSA, which corresponds to the 1:1 conjugation ratio.

To achieve a PA imaging modality implementation, traditionally, materials with high absorbance in the wavelength range of biological window of transparency and consequence thermal expansion for pressure sound production are used. In our case, gold nanocages (AuNCs) were chosen as functional additives embedded in the microbubble shell. Basically, the size of gold nanocages may vary depending on the synthetic methodology. The nanocages’ size and silver content also determine the optical properties of gold colloids: from visible to near-infrared extinction maxima. For the successful application of PA imaging in the first biological window (650–1350 nm), AuNCs with a maximum extinction peak at 780 nm were chosen to prepare. The morphology of the obtained nanoparticles was characterized by transmission electron microscopy and nanoparticles tracking analysis (NTA). The TEM micrograph of AuNCs provided in Figure 1b demonstrates their cubic shape with a side length of about 40 nm. Alongside, the size of AuNCs was determined by two independent methods: dynamic light scattering (DLS) and NTA. Characterization results are shown in Table 1; the sizes of 46 ± 3 nm measured by DLS and 50 ± 12 by NTA are consistent with the TEM data.

The implementation of gold nanoparticles into the microbubbles shell becomes possible only if their surface is coated with the same biocompatible polymer as the chosen material for microbubbles preparation. Thus, the coating of AuNCs with BSA was achieved by the simple ligand exchange of monovalent CTAB to multivalent protein at alkaline conditions (pH = 12). The chosen coating procedure is considered to be aggregation-free, and the complete removal of CTAB is anticipated. The TEM microphotograph at Figure 1c proves that the functionalization of the gold surface with BSA molecules does not affect both nanocages’ shape and size. Moreover, the parameters of the coating methodology were chosen correctly, since neither the size nor the concentration of nanoparticles seem to be changed during their coating with protein. Therefore, the size of AuNC-BSA was found to be 65 ± 10 nm by DLS and 64 ± 16 by NTA, and the solutions with the nanoparticles concentration of (8.5 ± 0.6) × 10^10^ particles/mL were used further without dilutions.

Additionally, with the use of DLS, the electrokinetic surface potential was determined for CTAB-coated and BSA-coated gold nanocages (Table 1). Successful coating with BSA was confirmed by changing the AuNCs surface potential from positive potential (+22 ± 2 mV) determined by CTAB, used as a capping agent during the initial synthesis, to the characteristic negative potential of BSA at pH = 6 (−15 ± 1 mV) [52].

### 3.2. Microbubbles Preparation and Characterization: Size, Concentration, and Stability

The sonication method was chosen for microbubbles preparation due to its simplicity and widespread use [53]. Air-filled microbubbles were obtained in saline solutions to track their properties in conditions close to physiological and to increase their compatibility. To understand how adding of various compounds in stabilizing shell affects microbubbles properties, the following four types of microbubbles were synthesized: based on pure BSA (BSA MBs), containing ZnPc (BSA-ZnPc MBs), containing AuNCs (BSA-Au MBs), and complicated structures containing both ZnPc and AuNCs (BSA-Au-ZnPc MBs).

**Figure 2:**
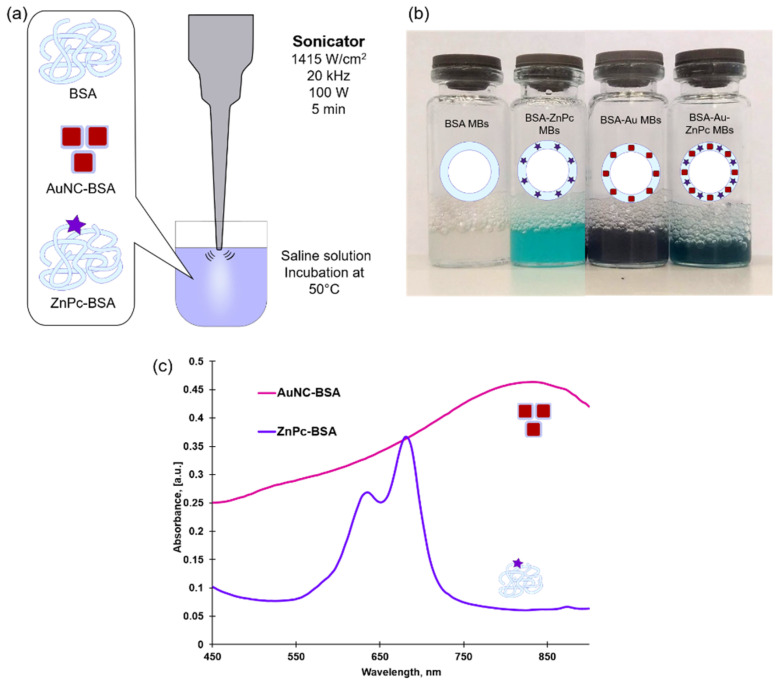
(**a**) Sonication method of microbubbles preparation. Bovine serum albumin (BSA) is used as the main component of microbubbles’ shells. BSA-coated gold nanocages (AuNC-BSA) and BSA-conjugated photodynamic dye zinc phthalocyanine (ZnPc-BSA) were used as functional additives. Dissolved in saline, solutions were incubated at 50 °C and sonicated for 5 min. (**b**) Photographs of resulting microbubbles solutions of different content: with BSA shell (BSA MBs), stabilized with ZnPc (BSA-ZnPC MBs), stabilized with Au NCs (BSA-Au MBs), and with both additives at the same time (BSA-Au-ZnPc MBs); (**c**) absorption spectra of BSA-coated gold nanocages (AuNC-BSA) and BSA-conjugated zinc phthalocyanine (ZnPc-BSA).

Microbubbles were prepared by the reproducible sonication method modified and approved in our laboratory to synthesize bubbles of different content [14,18]. Schematically, the ultrasound-assisted method is shown in Figure 2a. Briefly, a saline solution consisting of BSA, AuNC-BSA nanoparticles, or/and ZnPc-BSA conjugate additives was incubated at 50 °C to partially denature intramolecular disulfide bonds. Further sonication at maximum amplitude for 5 min of solutions results in BSA-stabilized microbubbles formation. Immediately after ultrasonic treatment, the solutions were placed in a refrigerator for 30 min (4 °C). Figure 2b represents solutions of obtained microbubbles of different compositions, and the opaqueness of solutions indicates the successful formation of microbubbles.

**Figure 3:**
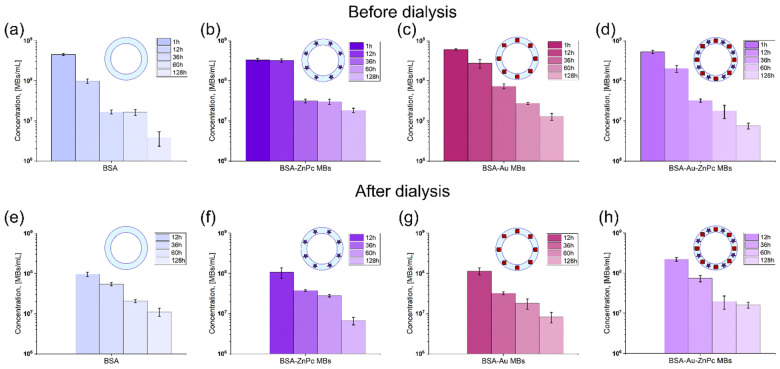
Microbubbles initial concentrations and their stability during storage without dialysis at 1, 12, and 36, 60 and 128 h after preparation: (**a**) microbubbles with the BSA shell only (BSA MBs), (**b**) microbubbles functionalized with ZnPc (BSA-ZnPc MBs), (**c**) microbubbles functionalized with AuNCs (BSA-Au MBs), (**d**) microbubbles stabilized with AuNCs and ZnPc (BSA-Au-ZnPc MBs); and for dialyzed probes at 12, 36, 60, and 60 h after preparation: (**e**) microbubbles with the BSA shell only (BSA MBs), (**f**) microbubbles functionalized with ZnPc (BSA-ZnPc MBs), (**g**) microbubbles functionalized with AuNCs (BSA-Au MBs), (**h**) microbubbles stabilized with AuNCs and ZnPc (BSA-Au-ZnPc MBs). All samples were stored at 4 °C. For convenience, concentration values are provided on a logarithmic scale.

To wash the prepared solutions from protein molecules not involved in the microbubble shell formation, they were dialyzed for 12 h against a saline solution. The concentration for each sample, with and without dialysis, was evaluated with a cell blood counter using optical microscopy (OM) images. All samples were stored at similar conditions (4 °C) to measure the stability of probes. The concentration of samples without dialysis was evaluated at 1, 12, and 36, 60, and 128 h after preparation and for dialyzed probes stored for 12, 36, 60, and 60 h after preparation. The microbubbles concentration decaying with the time of storage is presented in Figure 3.

In ascending order of the microbubbles’ concentration at 1 h after preparation, the samples are arranged as follows: BSA-ZnPc MBs (3.3 × 10^8^ MBs/mL), BSA MBs (4.5 × 10^8^ MBs/mL), BSA-Au-ZnPc MBs (5.2 × 10^8^ MBs/mL), BSA-Au MBs (6.0 × 10^8^ MBs/mL). The incorporation of dye to the bubble shell decreased the initial concentration of bubbles compared to the shell consisting of BSA only, while the gold-nanocage embedding led to a 1.3-fold increase in bubbles concentration. Unsurprisingly, simultaneous incorporation of photodynamic dye and nanoparticles also increases the concentration of prepared microbubbles compared to BSA MBs but less than with only gold nanocages addition. That indicates a greater contribution of gold nanocages on microbubbles stability during MBs formation than the zinc phthalocyanine influence. However, it turns out that initial concentration does not determine microbubbles’ stability over storage time. It can be seen that BSA MBs are less stable than any MBs with additives. Meanwhile, microbubbles containing ZnPc were the most stable sample, based on approximately the same concentration pairwise at 1 and 12 h after preparation and 36 and 60 h after preparation. The stability of gold-containing microbubbles (BSA-Au MBs and BSA-Au-ZnPc MBs) seems to be determined mainly by the presence of gold since both dependencies possess almost the same tendency and slope of decreasing. However, incorporating ZnPc into Au-containing MBs shell negatively affects their stability: the concentration of BSA-Au-ZnPc MBs reduces by 70 times over one week of storage compared to 47-fold decreasing for BSA-Au MBs by the same time. The embedding of gold nanocages increases the average molecular weight of the microbubble’s shell; that is why BSA-Au MBs possess lower initial concentration (too large weight is not stable) but higher storage stability, while the addition of the zinc phthalocyanine to the system enhances the system stability owing to the hydrophobicity of molecules and the resulting lower surface tension.

Microbubbles were washed using dialysis against the saline solution to get rid of BSA and ZnPc-BSA molecules that did not incorporate into the shell structure. AuNC-BSA conjugates are not washed out through the pores of the dialysis tube due to the significant molecular weight but precipitate on the tube’s inner wall. However, the dialysis procedure itself can influence the sample’s stability, and the presence of unbound additives in the solution may play a significant role in the stabilization of the microbubbles. After 12 h of dialysis, the microbubbles samples can be arranged in ascending order as follows: BSA MBs (9.6 × 10^7^ MBs/mL), BSA-ZnPc MBs (1.1 × 10^8^ MBs/mL), BSA-Au MBs (1.2 × 10^8^ MBs/mL), BSA-Au-ZnPc MBs (2.2 × 10^8^ MBs/mL). Hence, the simultaneous inclusion of dye and nanocages results in the most significant survival of MBs during the dialysis. Interestingly, when compared to the concentration of non-washed microbubbles after 12 h of storage, it turns out that the dialysis procedure does not contribute to the BSA-Au-ZnPc MBs concentration since it is the same for both washed and unwashed samples (2.2 × 10^8^ and 2.0 × 10^8^, respectively). The same peculiarity is found for only BSA-containing bubble-micromotors: 9.8 × 10^7^ for unwashed samples and 9.6 × 10^7^ for washed samples at 12 h after preparation. Moreover, dialyzed BSA MBs and BSA-Au-ZnPc MBs demonstrated even higher stability over storage time and final concentration one week after preparation than the same unwashed samples. Surprisingly, the most stable sample before dialysis (BSA-ZnPc MBs) does not show the same behavior when dialyzed and even becomes the less stable solution compared to the rest. Unbound ZnPc-BSA molecules floating near bubbles contribute to the stability along with being incorporated into the shell molecules, somehow preventing the bubble from shrinking and bursting.

Additionally, the amount of AuNCs in the microbubbles shell was evaluated. The concentration of Au-BSA nanoparticles was determined by NTA, and extinction spectra were measured for the solution used for microbubbles preparation and solution of dialyzed microbubbles of concentration 10^8^ MBs/mL. Assuming a uniform distribution of gold nanoparticles over microbubble and linear dependence of absorbance versus concentration in that range of AuNCs concentration, the number of nanoparticles per one bubble was found to be 172 ± 31. Assuming that the average diameter of the obtained microbubbles is equal to 1 μm, and, knowing from the literature data, the shell thickness of bubble with the protein shell can be considered 30 nm [16], and the volume of the shell is approximately 8.9 × 10^−2^ μm^3^. According to the DLS measurements, the volume of gold nanoparticles can be calculated from the assumption that a nanocage represents an ideal cube with a side length of 50 nm. Hence, the total volume occupied by gold nanocages within a shell equals 2.1 × 10^−2^ μm^3^. Thus, the filling factor, which may be implied as a ratio of the shell volume occupied by nanoparticles to the total volume, is roughly 0.25. That indicates reasonable and successful incorporation of gold nanocages to the microbubbles shell.

**Figure 4:**
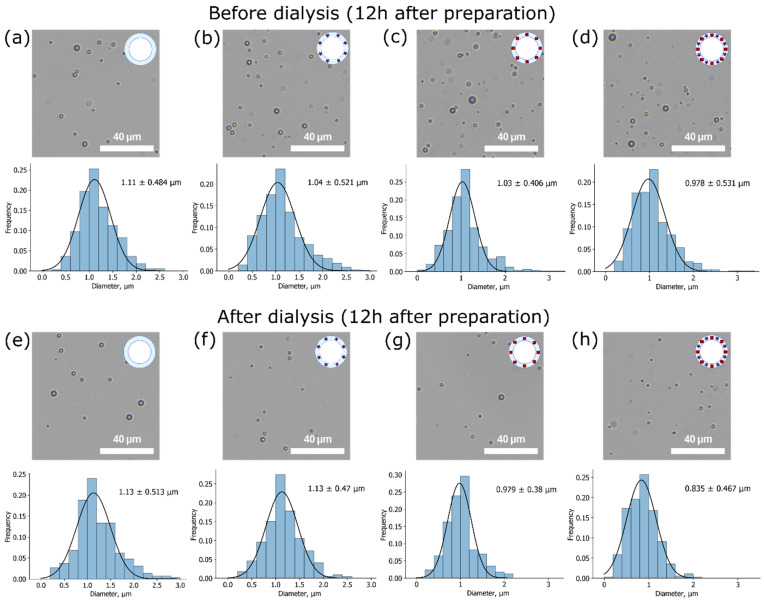
Optical microscopy (OM) images and microbubbles size distributions at 12 h after the preparation. Before dialysis: (**a**) BSA MBs, (**b**) BSA-ZnPc MBs, (**c**) BSA-Au MBs, (**d**) BSA-Au-ZnPc MBs; after dialysis: (**e**) BSA MBs, (**f**) BSA-ZnPc MBs, (**g**) BSA-Au MBs, (**h**) BSA-Au-ZnPc MBs.

As the size of microbubbles plays a crucial role in their possible applications, the size distributions were studied. Mean size measurements based on OM images for both unwashed and washed samples were made 1 and 12 h after sample preparation. OM images and mean size measurements for samples 12 h after preparation are presented in Figure 4. The normal Gaussian distribution of sizes was observed for all types of samples.

It can be seen that all microbubbles possess a micron mean size with a relatively narrow distribution as expected for chosen microbubbles production method, so they meet one of the main requirements for contrast agents to be less than 7 μm in diameter to pass lungs capillary successfully [17]. In descending order, not dialyzed samples stored for 12 h at 4 °C can be arranged as follows: BSA MBs (1.1 ± 0.5 µm), BSA-ZnPc MBs (1.0 ± 0.5 µm), BSA-Au MBs (1.0 ± 0.4 µm), BSA-ZnPc-AuNPs MBs (1.0 ± 0.5 µm). Since these calculated mean sizes coincide with the mean sizes of freshly prepared (1 h) samples (Figure S1), we can conclude that all samples except BSA-Au-ZnPc MBs demonstrated great stability in diameter during the first 12 h of storage. Hence, the uniform bursting of microbubbles of different sizes can be considered. In the case of two additives at the same time (BSA-Au-ZnPc MBs), the mean size increases during the first 12 h after preparation, which can be explained by Ostwald ripening as a result of high internal Laplace pressure of smaller microbubbles.

Samples after dialysis can be arranged in the same order: BSA MBs (1.1 ± 0.5 µm), BSA-ZnPc MBs (1.1 ± 0.5 µm), BSA-Au MBs (1.0 ± 0.4 µm), BSA-ZnPc-AuNPs MBs (0.8 ± 0.5 µm). Additionally, the statistical significance of the change in the diameter of the bubble-micromotors during the dialysis was checked using the Student’s t-test. If the calculated p-value is below 0.05, then the null hypothesis that the dialysis does not affect the diameter is rejected in favor of its significant effect. Therefore, the influence of dialysis on bubble size was found to be significant only for gold-containing samples (BSA-Au MBs and BSA-Au-ZnPc MBs). The remarkable decrease in the diameter of these samples can be related to the instability of big gold-based microbubbles with a relatively high molecular weight of the shell when in contact with dialysis tubes’ walls.

Overall, dialyzed BSA-Au-ZnPc MBs combine the highest survival during washing and optimal stability in concentration over the storage time and the smallest diameter that opens a good possibility for further study and use in biomedical applications.

### 3.3. Imaging Properties Characterization: Ultrasound, Optoacoustic, and Fluorescence Modalities

**Figure 5:**
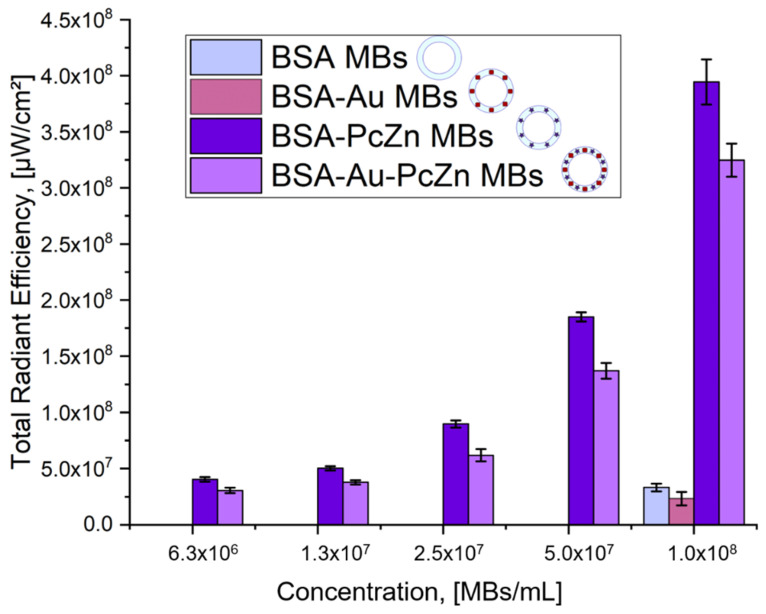
Total radiation efficiency calculated based on fluorescence imaging of samples: BSA MBs, BSA-Au MBs, BSA-ZnPc MBs, and BSA-Au-ZnPc MBs at excitation/emission pair of 675/720 nm at different bubble-micromotors concentrations.

The multimodal application potential of the microbubbles was realized by a combination of US imaging due to microbubbles’ relevant echogenicity properties, PA imaging due to the inclusion of AuNCs, and FL tomography due to the presence of photodynamic dye in the shell structure with the possibility to achieve PDT enhancement. These potential applications were tested for all prepared samples. The results of the FL tomography measurements depending on the microbubbles concentration and, as a result, the concentration of the photodynamic dye in the samples are shown in Figure 5. Fluorescence tomography measurements were conducted only for dialyzed microbubbles (12 h after preparation) to ensure that unbound dye molecules signal did not interfere with dye incorporated into the microbubbles shell. Both BSA-ZnPc MBs and BSA-Au-ZnPc MBs revealed high fluorescence intensity at 675/720 nm excitation/emission pair, while BSA MBs and BSA-Au MBs did not exhibit comparable fluorescent signals.

Fluorescence quenching was observed for the samples of microbubbles containing gold nanocages compared to those without nanoparticles (BSA MBs and BSA-ZnPc MBs). It can be observed that the incorporation of gold nanocages into the bubble shell modified with dyes (BSA-Au-ZnPc MBs) decreases the fluorescent signal of BSA-ZnPc MBs by 1.2 times. Various models of fluorescence quenching due to gold–fluorophore interactions were described in the literature [54], but predominantly, it is connected with overlapping gold nanoparticles plasmon resonance and emission band of fluorescent dye.

**Figure 6:**
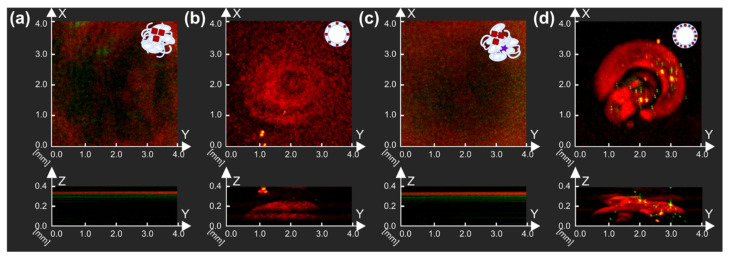
Raster-scanning optoacoustic mesoscopy measurements of (**a**) Au-BSA solution, (**b**) BSA-Au MBs, (**c**) ZnPc-BSA and Au-BSA solution, and (**d**) BSA-Au-ZnPc MBs. Projections of measurements in X and Y, X, and Z axes are presented.

Next, AuNCs samples were tested for their potential application in PA imaging since gold nanoparticles were already involved in research as promising PA contrast agents. Thus, AuNPs-containing MBs (BSA-Au MBs and BSA-Au-ZnPc MBs) and corresponding solutions used for microbubbles preparation (BSA and Au-BSA containing solution; BSA, ZnPc-BSA, and Au-BSA consisting solution) were studied by raster-scanning optoacoustic mesoscopy (RSOM), as one can see in Figure 6.

The presence of nanoparticles in BSA-Au MBs and BSA-Au-ZnPc MBs can be seen by their localization in the upper part of samples due to bubbles floating. BSA-Au-ZnPc MBs sample, which contained both AuNCs and complex with photodynamic dye (ZnPc-BSA) in the bubbles shell, demonstrated a higher PA response during measurements than microbubbles where the shell was stabilized by AuNCs only (BSA-Au MBs); thus, the highest PA signal corresponded to the BSA-Au-ZnPc MBs sample. Compared with initial solutions, bubbles-containing samples demonstrated higher PA signals, which can be described with the relevant properties demonstrated by the presence of microbubbles. Thus, the frequency of 33 MHz was validated as the optimal for PA imaging modality and suggested for further characterization in US imaging.

The absorption spectra of the dialyzed microbubbles were measured; as can be seen in Figure S2, all bubbles-containing samples had comparable extinction properties at the wavelength of 532 nm used for photoacoustic imaging characterization. Curiously, when the fluorescent dye is incorporated into shells already modified with gold nanoparticles, the extinction plasmon resonance peak of gold nanoparticles decreases, while the intensity of two peaks associated with dye enhances. Assuming the equal concentrations of nanoparticles and dyes in all kinds of microbubbles, this fact may be related to the certain interaction between gold nanoparticles and phthalocyanine. Apparently, phthalocyanine molecules coordinate around the gold nanocages, resulting in the lower contribution of gold surface electrons into the plasmon resonance effect as already described in the literature [55].

**Figure 7:**
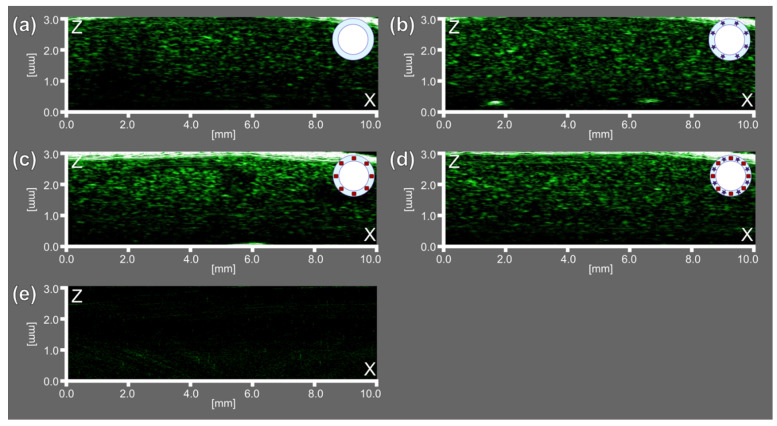
Ultrasound (US) imaging at a frequency of 33 MHz for (**a**) BSA MBs, (**b**) BSA-ZnPc MBs, (**c**) BSA-Au MBs, (**d**) BSA-Au-ZnPc MBs, and (**e**) saline solution.

Then, US imaging characterization at a frequency of 33 MHz revealed the possibility of using all bubbles-containing samples as US contrast agents since they revealed a significant acoustic response compared with saline only, which showed no acoustic response, as can be seen in Figure 7.

Thus, the gaseous core of microbubbles demonstrated acoustic properties relevant for their response in US imaging, while functional additives as AuNCs and ZnPc were relevant to achieve PA and FL imaging purposes, respectively. The sample consisted of microbubbles both with AuNCs and ZnPc-BSA additives in the shell (BSA-Au-ZnPc MBs) demonstrated the possibility of using trimodal (FL/PA/US) imaging, opening up the possibility of theranostics applications based on the use of microbubbles.

## 4. Conclusions

In this work, we report studies on the synthesis, characterization, and stability of bovine serum albumin (BSA)-based microbubbles that can be used as ultrasound contrast agents. To achieve additional imaging modalities and therapeutic functionalities along with ultrasound response, photodynamic dye (zinc phthalocyanine, ZnPc) and nanoparticles (gold nanocages, AuNCs) were incorporated into the microbubbles shell separately and simultaneously. ZnPc was covalently bound to BSA via carbodiimide synthesis for the further successful incorporation in the bubble structure, and MALDI-TOF proved the covalent nature of bonding. Gold nanocages stabilized by CTAB with the strong extinction peak at 780 nm were prepared (46 ± 3 nm, (8.1 ± 0.6) × 10^10^ particles/mL) and then coated with BSA for enhancing the biocompatibility and facile implementation of gold nanoparticles into the shell. The final parameters of the AuNC-BSA solution were as follows: 65 ± 10 nm, (8.5 ± 0.6) × 10^10^ particles/mL.

The successful incorporation of various additives (photodynamic dye and gold nanoparticles) for multimodal imaging was observed using the standard sonication method of microbubbles preparation. Regardless of the shell composition of microbubbles, stable dispersions were produced with a narrow size distribution with an average size of 1.0 ± 0.5 μm. In ascending order of the microbubbles’ concentration at 1 h after preparation, the samples are arranged as follows: BSA-ZnPc MBs (3.3 × 10^8^ MBs/mL), BSA MBs (4.5 × 10^8^ MBs/mL), BSA-Au-ZnPc MBs (5.2 × 10^8^ MBs/mL), BSA-Au MBs (6.0 × 10^8^ MBs/mL). Microbubbles were washed using dialysis against the saline solution to get rid of unreacted molecules that did not incorporate the shell structure. After 12 h of dialysis, the microbubbles samples are arranged in ascending order as follows: BSA MBs (9.6 × 10^7^ MBs/mL), BSA-ZnPc MBs (1.1 × 10^8^ MBs/mL), BSA-Au MBs (1.2 × 10^8^ MBs/mL), BSA-Au-ZnPc MBs (2.2 × 10^8^ MBs/mL). Hence, the initial concentration and the stability over dialysis procedure as well as over storage time of bi- and trimodal contrast agents were found to be higher than that of BSA-only microbubbles. The main reason for it is that the addition of zinc phthalocyanine to the system enhances its stability owing to the hydrophobicity of molecules and the resulting lower surface tension. At the same time, the embedding of gold nanocages increases the average molecular weight of the microbubble’s shell, resulting in higher storage stability.

For all obtained microbubbles, the desired functionalities were investigated. All types of the prepared samples have exhibited significant ultrasound response at a frequency of 33 MHz, proving their potential for use in US imaging. The BSA-ZnPc sample achieved the most significant fluorescence signal; its total radiant efficiency reached 4.0 × 10^8^ μW/cm^2^. Compared with solutions for microbubbles preparation, all bubbles-containing samples demonstrated more intensive PA signals, and the highest photoacoustic (PA) signal corresponded to the BSA-Au-ZnPc MBs sample. Although the simultaneous embedding of dye and gold nanoparticles (BSA-Au-ZnPc MBs) results in slight fluorescence quenching, it increases the intensity of the photoacoustic signal, thus opening an opportunity for bimodal (PA/US) imaging capability along with photodynamic therapy using zinc phthalocyanine. BSA-ZnPc MBs can also be used as a bimodal contrast agent for simultaneous ultrasound and fluorescent imaging.

Overall, the inclusion of gold nanocages and zinc phthalocyanine in the albumin shell structure results in a sufficient level of demanded properties, and the prepared trimodal microbubbles could be further exploited in preclinical biomedical research.

## Figures and Tables

**Table 1:** The characterization of nanoparticles by dynamic light scattering (DLS) and nanoparticles tracking analysis (NTA).

Sample	Size by DLS, nm	Size by NTA, nm	Zeta Potential, mV	Concentration, Particles/mL
AuNCs	46 ± 3	50 ± 12	+22 ± 2	(8.1 ± 0.6) × 10^10^
AuNCs-BSA	65 ± 10	64 ± 16	−15 ± 1	(8.5 ± 0.6) × 10^10^

## Data Availability

Data are contained within the article and Supplementary Materials.

## Supplementary Materials

The following are available online at www.mdpi.com/article/10.3390/mi12101161/s1, Figure S1: Optical microscopy (OM) images and microbubbles size distributions of freshly prepared MBs (upper panel): (a) BSA MBs, (b) BSA-ZnPc MBs, (c) BSA-Au MBs, (d) BSA-Au-ZnPc MBs; 12 h after the preparation (middle panel): (e) BSA MBs, (f) BSA-ZnPc MBs, (g) BSA-Au MBs, (h) BSA-Au-ZnPc MBs; after 12 h of dialysis: (i) BSA MBs, (j) BSA-ZnPc MBs, (k) BSA-Au MBs, (l) BSA-Au-ZnPc MBs; Figure S2: The extinction spectra of the freshly prepared microbubbles in the concentrations of 100, 50, and 25 million of MBs per mL: (a) BSA MBs, (b) BSA-Au MBs, (c) BSA-ZnPc MBs, (d) BSA-Au-ZnPc MBs.
