# Supplementary material for: Air-Filled Microbubbles Based on Albumin Functionalized with Gold Nanocages and Zinc Phthalocyanine for Multimodal Imaging
Source: Micromachines (Basel). 2021 Sep 27;12(10):1161. doi: 10.3390/mi12101161 (PMC8537308; doi:10.3390/mi12101161)
Supplement: Supplementary file 1 [file micromachines-12-01161-s001.zip › micromachines-1381256-supplementary.pdf]

micromachines

MDPI

Supplementary Materials: Air-Filled Microbubbles Based on Albumin Functionalized with Gold Nanocages and Zinc Phthalocyanine for Multimodal Imaging

Elizaveta A. Maksimova [1,], Roman A. Barmin [1], Polina G. Rudakovskaya [1], Olga A. Sindeeva [1], Ekaterina S. Prikhozhdenko [2], Alexey M. Yashchenok [1], Boris N. Khlebtsov [3], Alexander A. Solovev [4], Gaoshan Huang [4], Yongfeng Mei [4], Krishna Kanti Dey [5] and Dmitry A. Gorin [1,*]

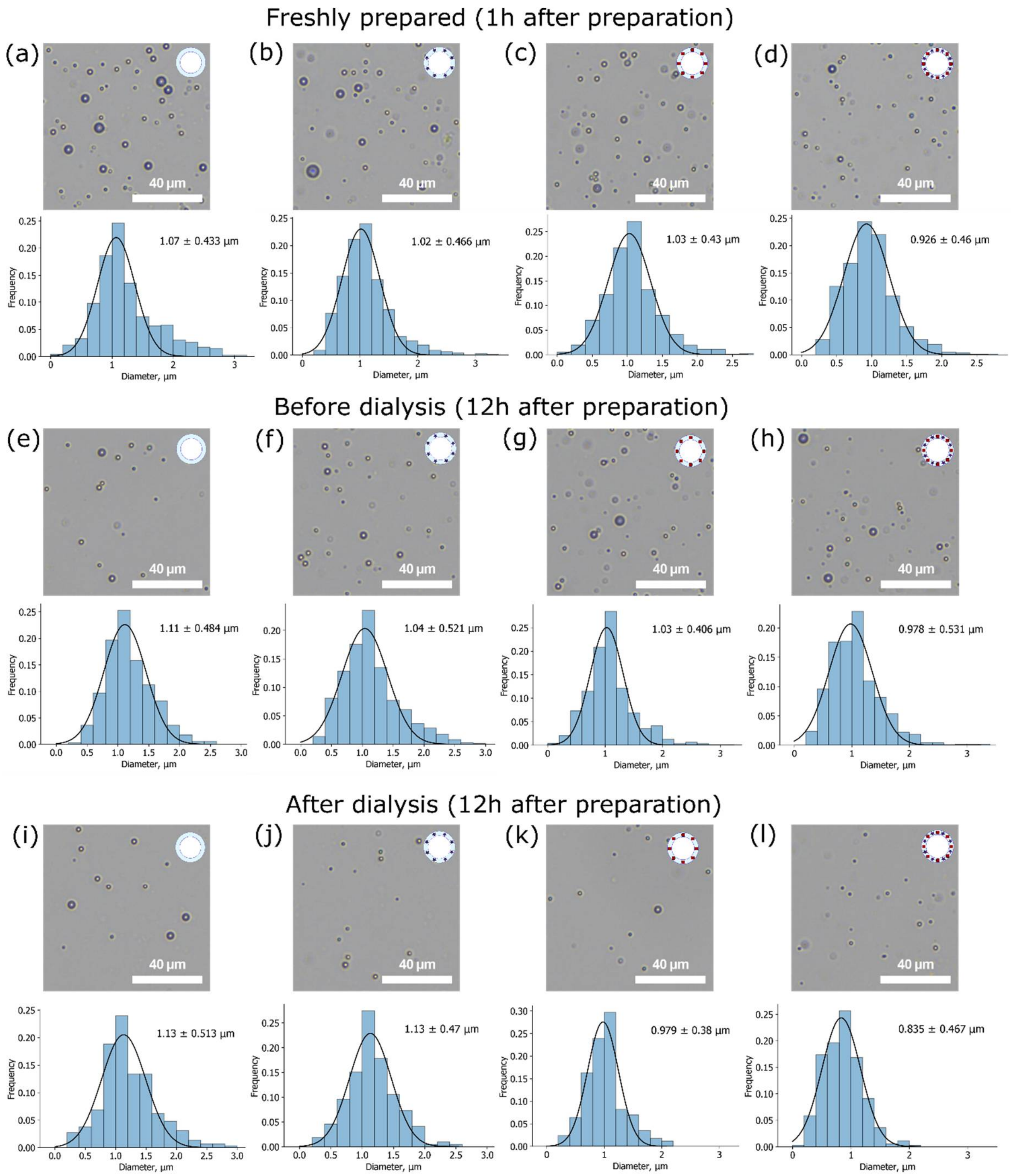

Figure S1. Optical microscopy (OM) images and microbubbles size distributions of freshly prepared MBs (upper panel): (**a**) BSA MBs, (**b**) BSA-ZnPc MBs, (**c**) BSA-Au MBs, (**d**) BSA-Au-ZnPc MBs; 12 hours after the preparation (middle panel):

(**e**) BSA MBs, (**f**) BSA-ZnPc MBs, (**g**) BSA-Au MBs, (**h**) BSA-Au-ZnPc MBs; after 12 hours of dialysis: (**i**) BSA MBs, (**j**) BSA-ZnPc MBs, (**k**) BSA-Au MBs, (**l**) BSA-Au-ZnPc MBs.

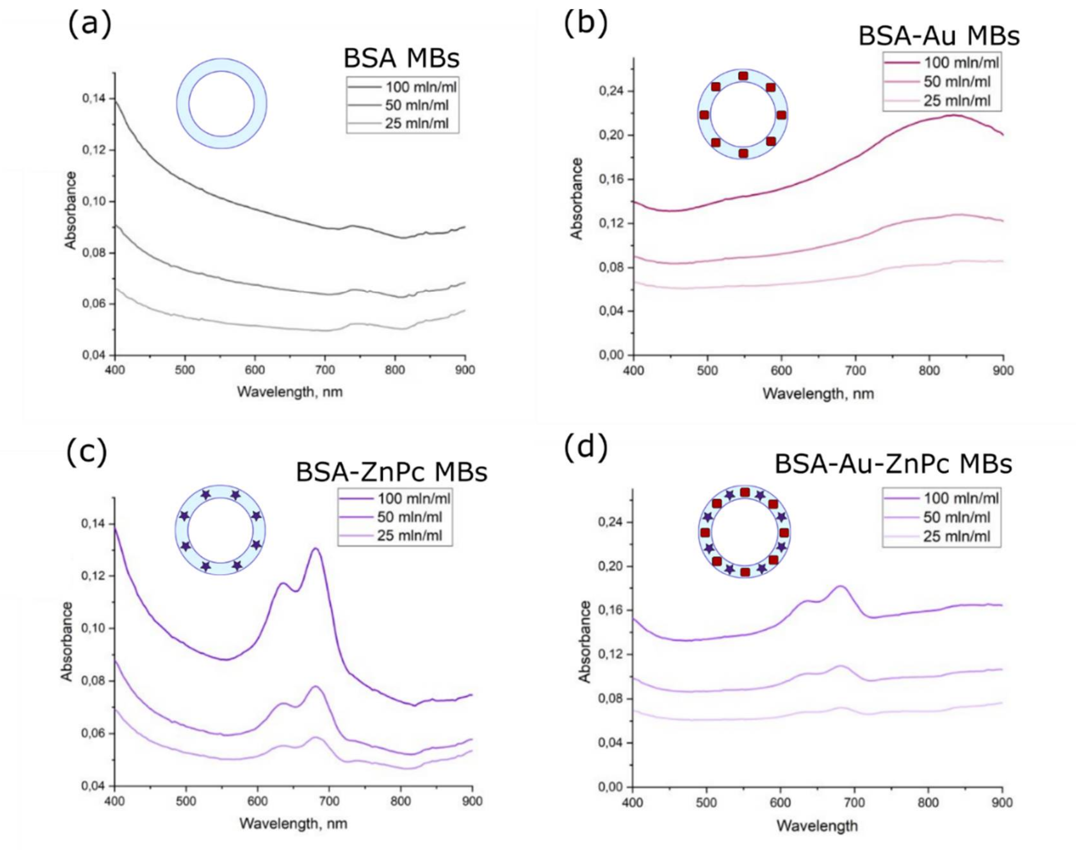

Figure S2. The extinction spectra of the freshly prepared MBs in the concentrations of 100, 50 and 25 million of MBs per mL: (**a**) BSA MBs, (**b**) BSA-Au MBs, (**c**) BSA-Zn MBs; (**d**) BSA-Au-ZnPc MBs.
